# Isovitexin alleviates acute gouty arthritis in rats by inhibiting inflammation via the TLR4/MyD88/NF-κB pathway

**DOI:** 10.1080/13880209.2021.1979595

**Published:** 2021-09-28

**Authors:** Xiujiang Sun, Peng Li, Xiaoyi Qu, Wenguang Liu

**Affiliations:** a Department of Orthopedics, Yantaishan Hospital, Yantai, China; b Department of Orthopedics and Traumatology, Dezhou Hospital of Traditional Chinese Medicine, Dezhou, China; c Clinical Teaching and Research Section, Yantai Nursing School, Yantai, China; d Department of Joint Surgery, the Second Hospital of Shandong University, Jinan, China

**Keywords:** Inflammatory factors, pathological changes, TLR4 inhibitor

## Abstract

**Context:**

The prevalence of gout has greatly increased, and it has become the most common inflammatory arthritis in men. Isovitexin possesses anti-inflammatory and antioxidant properties.

**Objective:**

We explored the effects of isovitexin on rats with acute gouty arthritis (GA).

**Materials and methods:**

Fifty-four Sprague-Dawley rats were assigned to five groups: sham, model, positive (colchicine, 0.3 mg/kg), isovitexin (100 mg/kg), TLR4 inhibitor (TAK-242, 3 mg/kg) and isovitexin + TAK-242. The gait of rats and the ankle joint swelling index were monitored. The levels of tumour necrosis factor-α (TNF-α), interleukin-1β (IL-1β) and IL-6, and pathological changes in the synovial tissues were determined.

**Results:**

Isovitexin significantly reduced the ankle joint swelling index at day 7 compared to that in the model group (4.39 ± 1.01 vs. 6.09 ± 1.31). Moreover, isovitexin alleviated the infiltration of inflammatory cells and ameliorated the proliferation of synovial cells. The levels of TNF-α (93.42 ± 5.02 pg/mL), IL-1β (25.46 ± 1.91 pg/mL) and IL-6 (194.71 ± 7.92 pg/mL) in the isovitexin group were significantly lower than in the model group (129.39 ± 5.43, 39.60 ± 2.71 and 223.77 ± 5.35 pg/mL). The expression of TLR4, MyD88 and p-NF-κB-p65 was remarkably decreased after isovitexin and colchicine treatment. The effect of isovitexin was similar to that colchicine. Furthermore, the combination of isovitexin and TAK-242 had better effect, and there was no significantly difference with colchicine treatment.

**Discussion and conclusions:**

Isovitexin ameliorates joint inflammation in acute GA via the TLR4/MyD88/NF-κB pathway. Isovitexin may be a potential substitute medicine for GA.

## Introduction

Gouty arthritis (GA), one type of arthritis, is caused by disorders in purine metabolism and uric acid excretion, which lead to the deposition of uric acid crystals in articular capsule, synovium, cartilage, bone and tissues around the joint. The incidence and prevalence rates of GA have been increasing every year (by about 1–2%), especially in developing countries (Lyu et al. 2019). GA is painful and leads to joint deformation and stiffness in the absence of prompt treatment. It can cause several complications, such as hypertension, diabetes and clinical coronary disease (van Walsem et al. 2015). Therefore, it is immensely significant to explore effective treatments for preventing or attenuating GA.

At present, the treatment of GA mainly includes non-steroidal anti-inflammatory drugs (NSAIDS), colchicine and glucocorticoids. However, these medications are not very clinically effective because of drug resistance, endogenous hormone suppression, gastrointestinal irritation and other adverse reactions (Mencherini et al. 2007; Nonaka et al. 2014; Simon Taylor 2017). The active extracts of natural herbs have increasingly attracted the attention of scientists owing to their promising curative effects, less adverse reactions and wide availability.

Isovitexin, an active flavonoid component of many traditional Chinese medicines, is a C-glycosylated derivative of apigenin and is widely distributed in many fruits and vegetables in nature. Increasing evidence indicates that isovitexin is involved in molecular functions related to anti-inflammatory and antioxidative activities (Zielińska-Pisklak et al. 2015; Lv et al. 2016; Liu et al. 2019). Isovitexin alleviates inflammatory response in the lipopolysaccharide (LPS)-activated RAW 264.7 macrophage cell line and inhibits inflammation in LPS-induced lung injury in mice (Lv et al. 2016). Recent results indicate that isovitexin could suppress osteoarthritis progression (Hu et al. 2021). Isovitexin has the advantages of easy extraction, high content and stable performance, which is expected to become an alternative drug for biodiversity (Liu et al. 2019). However, its effect on GA has not been reported. Colchicine is beneficial to the treatment of acute attack of gout. Previous studies have confirmed that the curative effect of low-dose colchicine is similar to that of high-dose colchicine, and before the relief of gout symptoms, gastrointestinal adverse events such as diarrhoea often occur at a high frequency (Janssens et al. 2008). In this study, we used colchicine as the positive group to investigate the effect of isovitexin.

Toll-like receptors (TLRs) play an important role in innate immunity. TLRs are effective activators of inflammatory response. Activation of TLRs can lead to the production of cytokines, chemokines, interferons and the transcription factor, NF-κB (Chen et al. 2018). It has been demonstrated that in primary GA the innate immune response can be activated by cellular pattern recognition receptor. Gout attack is related to the activation of type I transmembrane protein receptor, TLR4 and its downstream signalling pathway, by urate *in vivo*. The TLR4–MyD88–NF-κB–interleukin-1β (IL-1β) signalling pathway participates in the regulation of immune and inflammatory responses in GA (Liu-Bryan et al. 2005a, 2005b; Martin and Harper 2010; Mitroulis et al. 2013). However, there are few studies on the use of isovitexin in the treatment of gout. In this study, we analysed the effects of isovitexin on the content of inflammatory factors in peripheral blood of rats with acute GA. The data on the expression levels of TLR4 and MyD88, and on the phosphorylation of NF-κB-p65 in the synovium can help understand the therapeutic effect and anti-inflammatory mechanism of isovitexin on acute GA observed in this study, and should provide a theoretical basis for clinical treatment of GA.

## Materials and methods

### Animals

Fifty-four male Sprague-Dawley (SD) rats (weighing 160–180 g, Jinan Peng Yue Experimental Animal Breeding Co. Ltd. (Jinan, China); SCXK (Lu) 20140007) were maintained in standard steel wire cages under controlled conditions (temperature, 20–26 °C; relative humidity, 40–70%; illumination, 12 h light/dark cycle) with free access to food and water throughout the study. The study was approved by the Animal Care Committee of the Second Hospital of Shandong University, and all experimental procedures were conducted according to the National Institutes of Health (NIH) Guide for the Care and Use of Laboratory Animals.

### Animal models

The monosodium urate (MSU) crystal-induced rat GA model used in this study was the same as described in detail elsewhere with some alterations (Raucci et al. 2019). The rats were randomly assigned to one of the following five groups (nine rats in each group): sham, model, positive (colchicine, 0.3 mg/kg, Xishuangbanna Pharmaceutical Co., Ltd., Jinghong, China), isovitexin (100 mg/kg, Sigma-Aldrich, St. Louis, MO), TLR4 inhibitor (TAK-242, 3 mg/kg, Sigma-Aldrich, St. Louis, MO) and isovitexin (100 mg/kg)+TAK-242 (3 mg/kg). The GA model was induced using MSU crystals. All rats, including those in the control group, were anaesthetized by intraperitoneal injection of 1% pentobarbital sodium (40 mg/kg). After anaesthesia, the rats were fixed in supine position, their joints were depilated and sterilized with 75% ethanol, and 0.1 mL sodium urate solution was injected into the right posterior malleolus with a needle (diameter, 0.45 mm). The rat model of GA was established after three days. The diameter of the same part of the right posterior malleolus of rats before and after the development of model was measured and the swelling index was calculated, using the following equation: Swelling index=(ankle circumference before modelling – ankle circumference after modelling)/ankle circumference before modelling. After the model was built, colchicine gavage, isovitexin and TAK-242 were injected intraperitoneally once a day for 1 week. The model group was administered distilled water.

### ELISA assay

All the rats were anaesthetized and blood was collected from the abdominal aorta. The blood was allowed to clot and serum was obtained by centrifuging the blood at 3000×*g* for 10 min at 4 °C. The levels of IL-1β, IL-6 and tumour necrosis factor-α (TNF-α) were measured using respective ELISA kits (R&D System, Minneapolis, MN) in accordance with the manufacturer’s instructions.

### Haematoxylin and eosin (H&E) staining

The synovial tissue of the right ankle joint was quickly and carefully separated on ice and fixed in 4% paraformaldehyde at 4 °C for 24 h. The samples were embedded in paraffin and cut into 5 µm sections. The paraffin sections were dewaxed with xylene, dehydrated in serial dilutions of ethanol (the concentrations were 95%, 80% and 70%) and then stained with haematoxylin for 10 min and eosin (Beijing Solarbio Science & Technology Co., Ltd., Beijing, China) for 3 min at room temperature. The sections were then rinsed in distilled water for 30 s. The sections were washed two times (1 min each) with 95% anhydrous ethanol and three times in xylene (5 min each) and then mounted with Permount mounting medium (Thermo Fisher Scientific, Inc., Waltham, MA).

### Masson staining

In brief, the paraffin sections were dewaxed two times with xylene (5 min each), dehydrated two times with ethanol (10 min each) and then dehydrated in serial dilutions of ethanol (the ethanol concentrations were 95%, 90%, 80% and 70%) for 5 min. The sections were stained with haematoxylin for 5 min, eosin for 2 min and ponceau acid fuchsin solution (Nanjing Jianchen Bioengineering Institute, Nanjing, China) for 10 min, and then rinsed quickly rinsed with distilled water. The sections were then treated with an aqueous solution of phosphomolybdic acid for 5 min and stained with aniline blue solution for 5 min. Subsequently, they were treated with 1% glacial acetic acid for 1 min. The slices were treated twice with 95% ethanol, anhydrous ethanol and xylene, for 5 min each, and then mounted with Permount mounting medium (Thermo Fisher Scientific, Inc., Waltham, MA).

### Immunohistochemistry

The samples were embedded in paraffin and cut into 5 µm sections. The paraffin sections were dewaxed by dipping in xylene and anhydrous ethanol, two times for 5 min each, and then treated with serial dilutions of ethanol (the concentration were 95%, 80% and 70%) for 5 min, and rinsed with distilled water for 5 min. The sections were treated with Tris EDTA/citrate buffer in microwave oven for 10 min, cooled to room temperature, and then washed with phosphate-buffered saline (PBS) three times for 5 min each. Furthermore, the sections were treated with 3% H_2_O_2_ for 15 min, and then washed with PBS three times for 5 min each. The sections were incubated with 5% BSA block buffer at 37 °C for 30 min. The primary antibodies against TLR4 and NF-κB were added and overnight incubation was done at 4 °C. Thereafter, the sections were washed with PBS three times for 5 min each and subsequently incubated with horseradish peroxidase-labelled secondary antibody at 37 °C for 30 min. After incubation, the sections were washed with PBS three times for 5 min each, incubated with diamino-benzidine (DAB) for 5 min, and then rinsed with water. The sections were counterstained with haematoxylin for 1 min and washed in running water two times (3 min each). The slides were observed at ×200 magnification under an optical microscope (Olympus, Tokyo, Japan) and positively stained cells were counted using the Image J software (NIH, Bethesda, MD).

### QRT-PCR

Total RNA was extracted from the synovial tissue using TRIzol (Invitrogen, Carlsbad, CA). RNA was reverse transcribed to cDNA using the SuperScript III Reverse Transcriptase (Thermo Fisher Scientific, Waltham, MA). Quantitative real-time polymerase chain reaction (qRT-PCR) was performed on Mastercycler ep realplex2 (Eppendorf, Hamburg, Germany), under the following conditions: 95 °C for 2 min, 95 °C for 5 s and 60 °C for 30 s (35 cycles). The data were analysed using the 2^−ΔΔCt^ method and GAPDH mRNA was used as an internal control. The sequences of primers used are listed in Table 1.

**Table 1.** Primer sequences.

Gene	Primer sequence
TLR4	Forward 5′-CTCACAACTTCAGTGGCTGGATTTA-3′
	Reverse 5′-GTCTCCACAGCCACCAGATTCTC-3′
MyD88	Forward 5′-GAGATCCGCGAGTTTGAGAC-3′
	Reverse 5′-CTGTTTCTGCTGGTTGCGTA-3′
GAPDH	Forward 5′-ATGTTCGTCATG GGTGTGAA-3′
	Reverse 5′-TGTGGTCATGAGTCCTTCCA-3′

### Western blot analysis

The synovial tissue was collected and homogenized with the radioimmunoprecipitation assay (RIPA) buffer (Beyotime Biotechnology, Shanghai, China), centrifuged at 12,000×*g* at 4 °C for 10 min. The content of protein in the homogenate was determined using the bicinchoninic acid (BCA) kit (Beyotime, Shanghai, China). Protein sample (50 μg) was resolved by sodium dodecyl sulphate-polyacrylamide gels electrophoresis (SDS-PAGE). The protein was transferred to a polyvinylidene fluoride (PVDF) membrane (Millipore, Billerica, MA) using semi-drying transfer. The membrane was blocked by incubating with 5% skimmed milk for 2 h. Thereafter, primary antibody was added and an overnight incubation was done at 4 °C. The membrane was washed with Tris buffered saline Tween (TBST) buffer three times, and then incubated with horseradish peroxidase-labelled secondary antibody at 37 °C for 2 h. The protein bands were visualized using the ECL luminescent reagent (Roche, Basel, Switzerland). The bands were analysed densitometrically using the Image J software (NIH, Bethesda, MD). GAPDH was used as an internal control.

### Statistical analysis

Results are expressed as mean ± standard deviation (SD). Statistical analysis was performed with one-way analysis of variance (ANOVA) used for comparisons among the groups followed by least significant difference (LSD) *post hoc* test with the SPSS 19.0 statistical software (SPSS Inc., Chicago, IL). Values of *p* < 0.05 were regarded as statistically significant.

## Results

### Gait changes in rats

The right posterior malleolus of rats in the model groups showed obvious swelling, lameness or tripod gait. As shown in Table 2, compared with the gait score of rats in the sham group (2.24 ± 0.87), the scores of rats in other groups were significantly higher (*p* < 0.05) at day 7. The gait scores in the isovitexin (4.39 ± 1.01), TAK-242 (4.43 ± 1.04) and isovitexin + TAK-242 (4.09 ± 1.03) groups were decreased compared to the score in the model group (6.09 ± 1.31), while they were still higher than positive group (3.71 ± 0.98).

**Table 2.** Gait scores at different time points.

Group	1 day	3 days	5 days	7 days
Sham	2.03 ± 0.81	2.21 ± 0.74	2.11 ± 0.96	2.24 ± 0.87
Model	5.23 ± 1.12*	5.89 ± 1.56*	6.12 ± 1.48*	6.09 ± 1.31*
Positive	5.17 ± 1.53**	4.71 ± 1.34*^,#^	4.21 ± 1.18*^,##^	3.71 ± 0.98^##^
Isovitexin	5.21 ± 1.65**	5.09 ± 1.31*	4.82 ± 1.21*^,#^	4.39 ± 1.01*^,#,&^
TAK-242	5.26 ± 1.67**	5.03 ± 1.27*	4.81 ± 1.19*^,#^	4.43 ± 1.04*^,#,&^
Isovitexin + TAK-242	5.18 ± 1.69**	4.94 ± 1.48*	4.42 ± 1.23*^,##^	4.09 ± 1.03*^,##^

vs. sham group, *p < 0.05, **p < 0.01; vs. model group, ^#^*p* < 0.05, ^##^*p* < 0.01; vs. positive group, ^&^*p* < 0.05.

### Ankle swelling index

The joint swelling index of rats in the model, isovitexin, TAK-242 and isovitexin + TAK-242 groups was significantly increased compared with that in the sham group (*p* < 0.05, Table 3), indicating that the GA model was successfully induced. At seven days after treatment, the joint swelling index in the isovitexin, TAK-242 and isovitexin + TAK-242 groups were significantly decreased compared with that in the model group, and there was no significant difference compared to positive group (*p* > 0.05).

**Table 3.** Changes in the ankle swelling index at different time points.

Group	1 day	3 days	5 days	7 days
Sham	0.13 ± 0.02	0.14 ± 0.03	0.13 ± 0.03	0.13 ± 0.03
Model	0.32 ± 0.12*	0.48 ± 0.11*	0.49 ± 0.20*	0.51 ± 0.17*
Positive	0.31 ± 0.13*	0.34 ± 0.12*	0.22 ± 0.13*^,#^	0.18 ± 0.07*^,##^
Isovitexin	0.31 ± 0.14*	0.39 ± 0.09*	0.25 ± 0.12*^,#^	0.23 ± 0.09*^,##^
TAK-242	0.29 ± 0.11*	0.37 ± 0.09*	0.26 ± 0.12*^,#^	0.24 ± 0.09*^,##^
Isovitexin + TAK-242	0.28 ± 0.09*	0.35 ± 0.13*	0.23 ± 0.11*^,#^	0.21 ± 0.08*^,##^

vs. sham group, *p < 0.05 vs. model group, ^#^*p* < 0.05, ^##^*p* < 0.01.

### Pathological changes assessed by H&E staining

**Figure 1.**
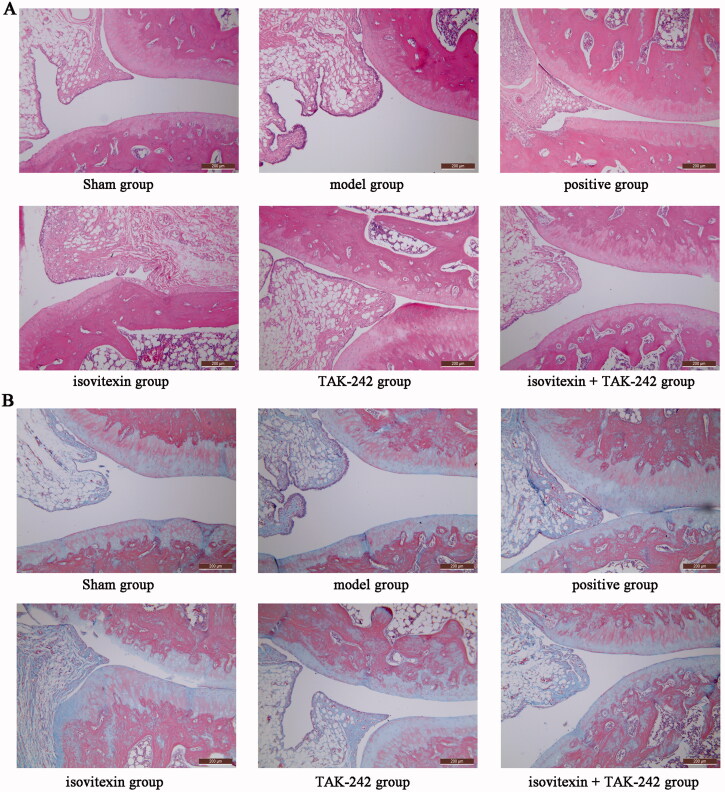
Effects of isovitexin on synovial tissues visualized using haematoxylin and eosin and Masson staining (×200). (A) Haematoxylin and eosin staining; (B) Masson staining.

The joint tissue structure was intact, and no infiltration of inflammatory cells was observed in the sham group (Figure 1(A)). In the model group, the joint tissue was severely damaged, the tissue structure was loose, some areas were necrotic and shed, the cells showed oedema and focal haemorrhage. Infiltration of a large number of inflammatory cells could be seen. In the isovitexin and TAK-242 groups, the joint tissue showed a neat joint structure, and cells were clear and regularly distributed. The infiltration of inflammatory cells was mainly in the outer layer, and rarely in the lower layer, and there were few eosinophilic granulocytes. The joint tissue in the isovitexin + TAK-242 and positive groups showed a neat tissue structure, tissue cells were clear and regularly distributed, and there was less infiltration of inflammatory cells.

The scores for each group are shown in Table 4. No damage was seen in the sham group. The histological damage score was not measured. The model group had the highest score. The score of the isovitexin + TAK-242 group was significantly lower than those of the isovitexin and TAK-242 groups (*p* < 0.05), and there was no significant difference compared to the positive group (*p* > 0.05).

**Table 4.** Joint tissue injury score.

Group	Score of tissue damage
Sham	0
Model	10.21 ± 1.09**
Positive	3.21 ± 1.42*^,##^
Isovitexin	6.07 ± 1.21*^,#,&^
TAK-242	6.29 ± 1.26*^,#,&^
Isovitexin + TAK-242	5.17 ± 1.54*^,#,^,★^

vs. sham group, *p < 0.05, **p < 0.01; vs. model group, ^#^*p* < 0.05, ^##^*p* < 0.01; vs. positive group, ^&^*p* < 0.05; vs. isovitexin group, ^^^*p* < 0.05; vs. TAK-242 group, ^★^*p* < 0.05.

### Changes observed in Masson staining

As shown in Figure 1(B), rats in the sham group had lower levels of inflammatory factors in the joint tissue, weak infiltration of the blue stain, and high tissue density. The level of inflammatory factors was high in the model group, the blue colour was seen to infiltrate the deep layers of the tissue, the density of the tissue was low, and the structure was loose. The inflammatory factors in the isovitexin and TAK-242 groups were obviously distributed on the surface layer of the tissue, there was little infiltration in the lower layer, and the tissue density was higher than in the model group. The inflammatory factors in the positive and isovitexin + TAK-242 groups showed infiltration, which was mainly distributed on the surface layer of the tissue, and the tissue density was high.

### Comparison of serum TNF-α, IL-1β and IL-6 content in different groups

**Figure 2.**
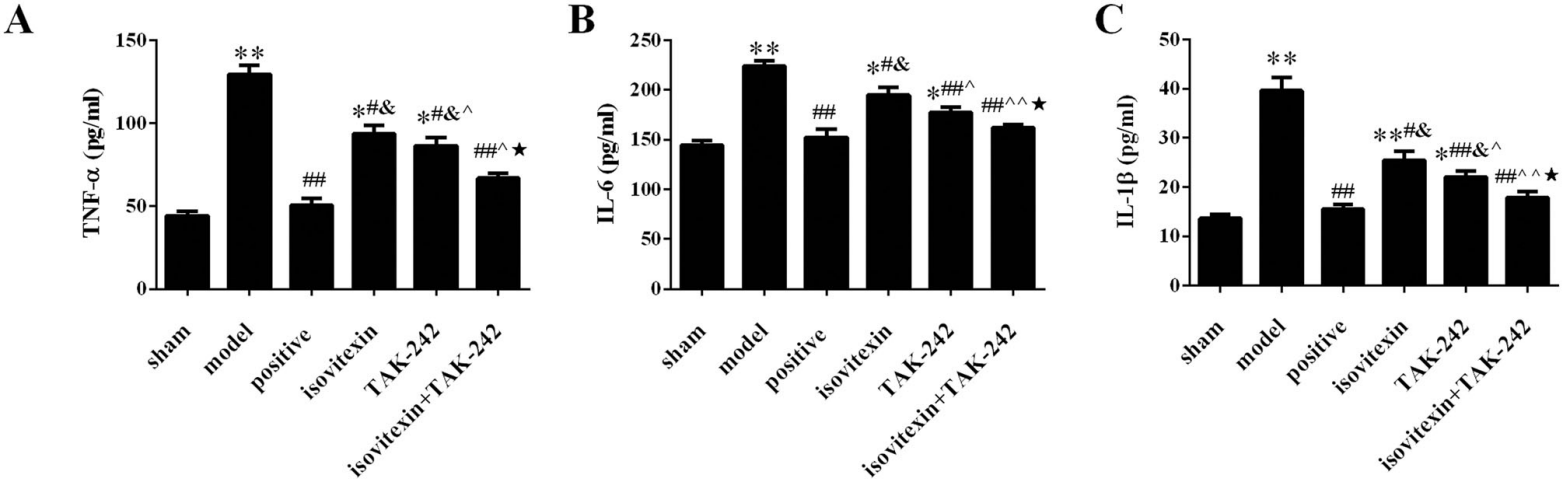
Comparison of the inflammation index in each group. Serum levels of (A) TNF-α; (B) IL-6; (C) IL-1β. *p < 0.05 compared with the sham group, **p < 0.01 compared with the sham group; ^#^*p* < 0.05 compared with the model group, ^##^*p* < 0.01 compared with the model group; ^&^*p* < 0.05 compared with the positive group; ^^^*p* < 0.05 compared with the isovitexin group, ^^^^*p* < 0.01 compared with the isovitexin group; ^★^*p* < 0.05 compared with the TAK-242 group.

Compared with the sham group, the content of TNF-α, IL-1β and IL-6 in the model group was increased (*p* < 0.05). After treatment, the levels of TNF-α, IL-1β and IL-6 were decreased in positive, isovitexin, TAK-242 and isovitexin + TAK-242 groups, there were significant differences with the model group (*p* < 0.05). The levels of TNF-α, IL-1β and IL-6 in the isovitexin + TAK-242 group were the lowest, and there was no significant difference compared with the levels in the sham and positive groups (*p* > 0.05, Figure 2).

### Comparison of the expression levels of TLR4 and MyD88 in the synovial tissue of rats

**Figure 3.**
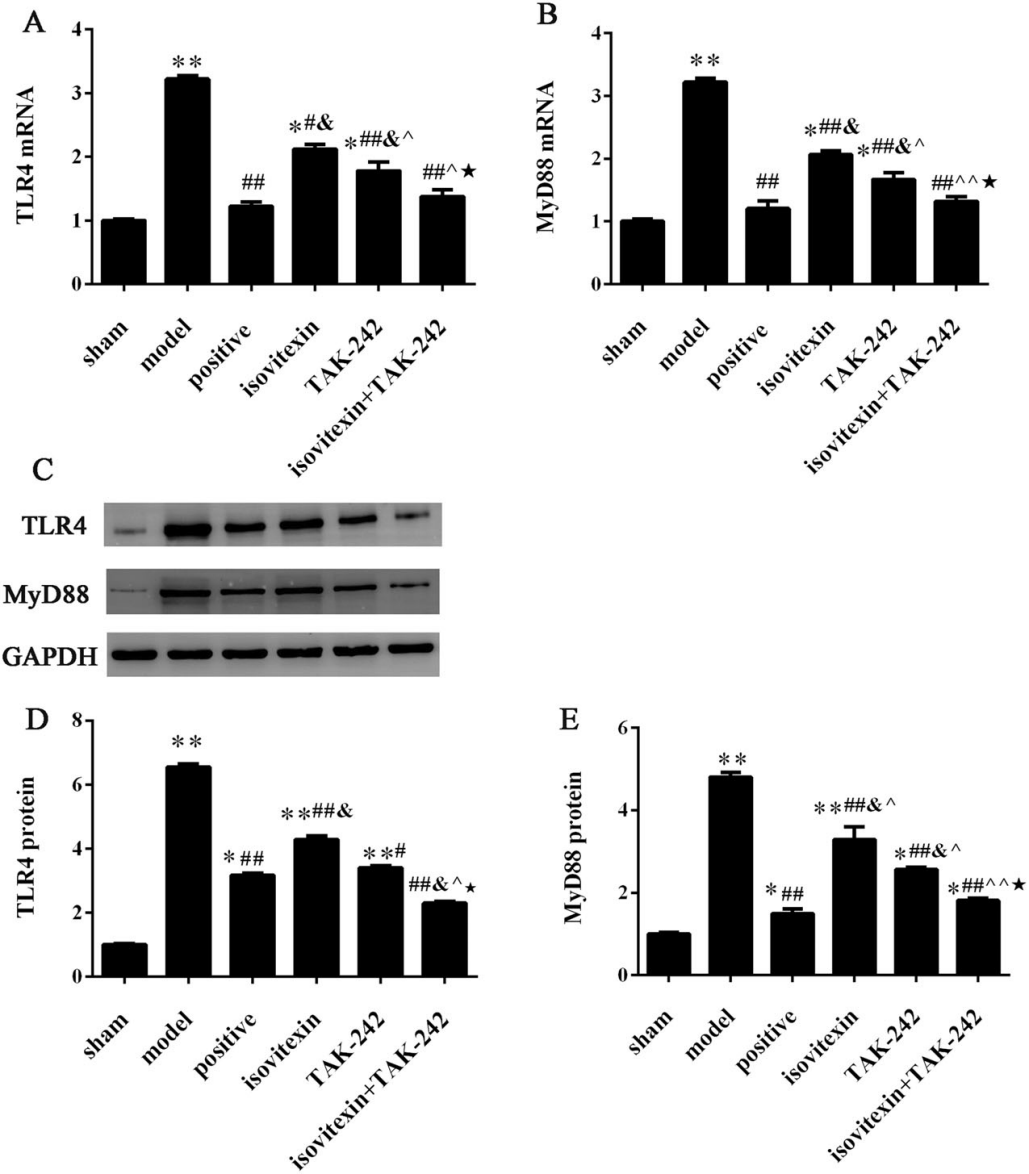
Expression of TLR4 and MyD88. Expression of (A) TLR4 mRNA. (B) MyD88 mRNA. (C) Representative images of western blot analysis. Relative protein expression levels of (D) of TLR4; (E) MyD88. *p < 0.05 compared with the sham group, **p < 0.01 compared with the sham group; ^#^*p* < 0.05 compared with the model group, ^##^*p* < 0.01 compared with the model group; ^&^*p* < 0.05 compared with the positive group; ^^^*p* < 0.05 compared with the isovitexin group, ^^^^*p* < 0.01 compared with the isovitexin group; ^★^*p* < 0.05 compared with the TAK-242 group.

The expression of TLR4 and MyD88 at the mRNA level was assessed by qRT-PCR and at the protein level by immunohistochemical and western blot analyses. The results of qRT-PCR and western blot analyses showed that the expression of TLR4 and MyD88 at the mRNA and protein levels in the model group was significantly increased compared with that in the control group (*p* < 0.05). After treatment, the mRNA and protein levels of TLR4 and MyD88 were decreased in the positive, isovitexin, TAK-242 and isovitexin + TAK-242 groups, and there was remarkable difference with the model group (*p* < 0.05, Figure 3).

**Figure 4.**
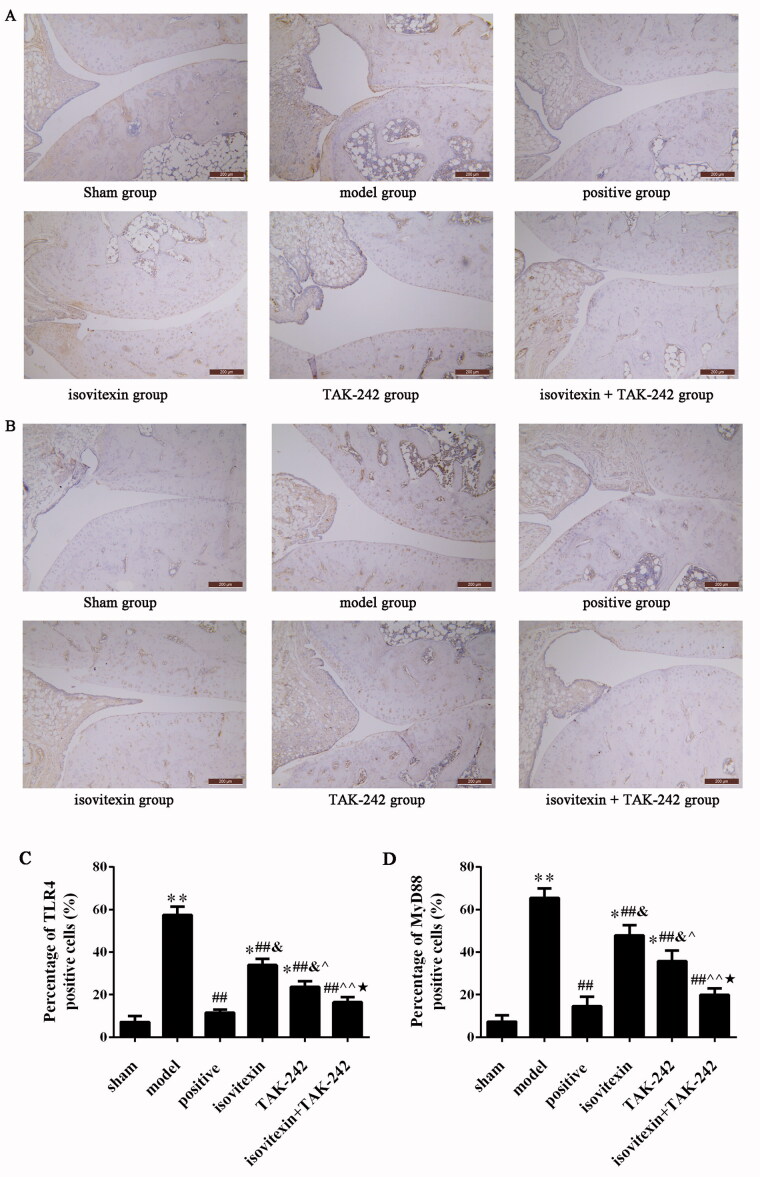
Immunohistochemical detection of TLR4 and MyD88 expression in the synovial tissue (magnification, ×200; scale bar: 200 μm). (A) Immunohistochemical analysis of TLR4 in each group. (B) Percentage of cells positive for TLR4. (C) Immunohistochemical analysis of MyD88 in each group. (D) Percentage of cells positive for MyD88. *p < 0.05 compared with the sham group, **p < 0.01 compared with the sham group; ^##^*p* < 0.01 compared with the model group; ^&^*p* < 0.05 compared with the positive group; ^^^*p* < 0.05 compared with the isovitexin group, ^^^^*p* < 0.01 compared with the isovitexin group; ^★^*p* < 0.05 compared with the TAK-242 group.

The results of immunohistochemical assay showed that the number of positive cells in the model group was increased compared with that in the control group (*p* < 0.05). After treatment with isovitexin and TAK-242, the number of positive cells was decreased in the positive, isovitexin, TAK-242 and isovitexin + TAK-242 groups (*p* < 0.05). The number of positive cells in the isovitexin + TAK-242 group was the lowest, and there was no significant difference compared with that in the sham and positive groups (*p* > 0.05, Figure 4). The results of the expression levels of TLR4 and MyD88 were consistent for the qRT-PCR and western blot analyses.

### Comparison of the expression levels of NF-κB p65 in the synovial tissue of rats

**Figure 5.**
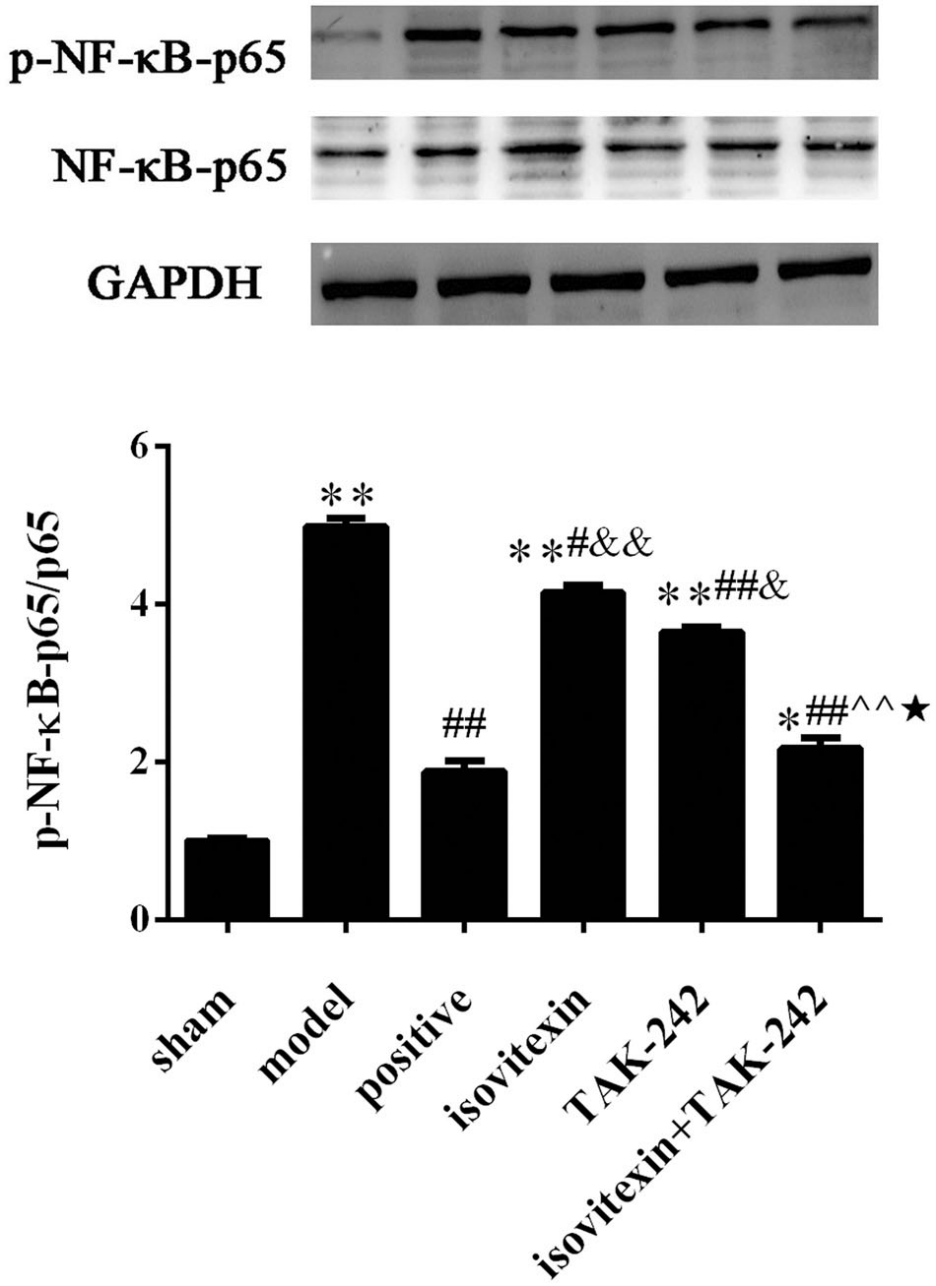
Level of p-NF-κB-p65. (A) Representative images of western blot analysis. (B) Relative protein expression levels of NF-κB. *p < 0.05 compared with the sham group, **p < 0.01 compared with the sham group; ^#^*p* < 0.05 compared with the model group, ^##^*p* < 0.01 compared with the model group; ^&^*p* < 0.05 compared with the positive group, ^&&^*p* < 0.01 compared with the positive group; ^^^^*p* < 0.01 compared with the isovitexin group; ^★^*p* < 0.05 compared with the TAK-242 group.

The expression of p-NF-κB p65 in the model group was increased compared with that in the sham group (*p* < 0.05). After treatment, the expression of p-NF-κB p65 was decreased in the isovitexin, TAK-242 and isovitexin + TAK-242 groups, and there were significantly differences with the expression in the model group (*p* < 0.05). The expression of p-NF-κB p65 in the isovitexin + TAK-242 group was the lowest (Figure 5).

## Discussion

When the level of serum uric acid in patients with GA increases, the supersaturated uric acid salt precipitates, forming crystals and deposits in the synovial tissue. Sodium urate is recognized by TLR4 and activates the TLR4 signal pathway, resulting in the transcription, synthesis, and release of a series of downstream pro-inflammatory factors, such as IL-1 β, which leads to acute inflammatory reaction in joints. This acute inflammatory attack is accompanied by a process of severe pain in which neutrophils enter the joint or the tissues around the joint, phagocytize the deposited MSU microcrystals, release inflammatory cytokines and mononuclear macrophage membranes dissolve, leading to joint swelling and skin reddening (Sabina et al. 2010). In this study, the swelling of the right ankle joint in the model group occurred gradually after the establishment of the model, and reached the peak at 72 h. The swelling index of the joint in the positive, isovitexin, TAK-242 and isovitexin + TAK-242 groups at each time point after the establishment of the model was significantly higher than that in the sham group, but was lower than in the model group, indicating that colchicine, and isovitexin can alleviate the swelling of joints.

TLR4 is mainly expressed on the surface of various immune cells, except T cells, B cells and NK cells. The severity of arthritis in TLR4-deficient mice has been reported to be significantly reduced (Shi et al. 2003). Qing et al. (2014) found that the expression of TLR4 and NF-κB in patients with acute GA was significantly higher than that in healthy people, which confirmed that TLR4 participates in the onset and development of acute inflammatory response in patients with gout. Clinical and experimental studies have confirmed that after activation, TLRs can induce a number of inflammatory factors, such as TNF-α, IL-1β, IL-6, which play important roles in the inflammatory process of acute gout (So et al. 2007; Kawai and Akira 2010; Cai et al. 2014). IL-1β is closely related to the production and intensity of inflammatory response in gout (Liu-Bryan et al. 2005b; Kawai and Akira 2010). In the present study, we observed an increase in the levels of TNF-α, IL-6 and IL-1β in the model group, which promoted the inflammation and proliferation of synovial cells. H&E and Masson staining also showed that the content of inflammatory factors in the model group was higher than in the sham group. After treatment, the levels of TNF-α, IL-6 and IL-1β in the positive, isovitexin, TAK-242 and isovitexin + TAK-242 groups were significantly reduced compared with the respective levels in the model group, and the reduction of the levels was more in the isovitexin + TAK-242 group, which verified that blocking of TLR4 can affect the production and release of important inflammatory cytokines that lead to acute gout attack. The effect of isovitexin + TAK-242 was similar to colchicine. Isovitexin can reduce the production and release of inflammatory cytokines, and thereby play an anti-inflammatory role.

NF-κB is a key downstream nuclear transcription factor at the hub of the TLR4 signalling pathway (Zhang et al. 2017). MSU crystals can directly activate the TLRs. The activated TLRs can form a complex through the C-terminal Toll/interleukin-1 receptor (TIR) domain of MyD88, and finally activate NF-κB (Wang et al. 2017). After NF-κB is activated, it starts the transcription of related genes, promotes the release of cytokines, which in turn can further activate NF-κB, thus forming a positive feedback loop, constantly enhancing the inflammatory response (Aderem and Ulevitch 2000). Previous study indicated that isovitexin suppresses NF-κB pathway in osteoarthritis (Hu et al. 2021). In our study, isovitexin can significantly inhibit the expression of TLR4 protein in the synovium of rats with acute gout, and can also significantly antagonize the expression of NF-κB p65 in the synovium. This suggests that isovitexin can inhibit the activation of NF-κB by weakening the recognition and internalization of TLR4 in inflammatory response, blocking the intracellular transduction with the MyD88 junction, which is consistent with previous experimental results. In addition, the isovitexin + TLR4 inhibitor treatment can enhance the inhibition of the activation of related inflammatory factors. From another point of view, isovitexin can indeed play an inhibitory role in the main TLR4/MyD88/NF-κB signal transmission pathway.

## Conclusions

The results of this study further prove that isovitexin can reduce the synovitis response in rats with acute gout. It downregulates the expression of TLR4 and MyD88, and affects the activation of the downstream signal element, NF-κB. The multi-target antagonism of TLR4/MyD88/NF-κB inflammatory response signal transduction pathway might be one of the anti-inflammatory mechanisms of TLR4/MyD88/NF-κB in the treatment of acute gout. The regulation of TLR4/MyD88/NF-κB inflammatory response signal transduction pathway plays an important role in its anti-inflammatory effect. However, the occurrence of inflammation is a complex pathological process involving multiple pathways, and the effect of traditional Chinese medicines is also characterized by multiple pathways and multiple targets. In this study, we only observed the changes in the expression of components of the TLR4/MyD88/NF-κB pathway. The mechanism underlying the anti-inflammatory effect of independent pathways, JNK, MAPK and other signalling pathways warrants further studies.

## References

[CIT0001] Aderem A, Ulevitch RJ. 2000. Toll-like receptors in the induction of the innate immune response. Nature. 406(6797):782–787. 10963608 10.1038/35021228

[CIT0002] Cai Y, Peng YH, Tang Z, Guo XL, Qing YF, Liang SH, Jiang H, Dang WT, Ma Q, He C, et al. 2014. Association of Toll-like receptor 2 polymorphisms with gout. Biomed Rep. 2(2):292–296. 24649113 10.3892/br.2014.224 PMC3917755

[CIT0003] Chen CY, Kao CL, Liu CM. 2018. The cancer prevention, anti-inflammatory and anti-oxidation of bioactive phytochemicals targeting the TLR4 signaling pathway. Int J Mol Sci. 19(9):2729. 10.3390/ijms19092729 PMC6164406 30213077

[CIT0004] Hu X, Li R, Sun M, Kong Y, Zhu H, Wang F, Wan Q. 2021. Isovitexin depresses osteoarthritis progression via the Nrf2/NF-κB pathway: an *in vitro* study. J Inflamm Res. 14:1403–1414. 33883918 10.2147/JIR.S299557 PMC8053716

[CIT0005] Janssens HJ, Lucassen PL, Van de Laar FA, Janssen M, Van de Lisdonk EH. 2008. Systemic corticosteroids for acute gout. Cochrane Database Syst Rev. 2(2):CD005521. 10.1002/14651858.CD005521.pub2 PMC8276233 18425920

[CIT0006] Kawai T, Akira S. 2010. The role of pattern-recognition receptors in innate immunity: update on Toll-like receptors. Nat Immunol. 11(5):373–384. 20404851 10.1038/ni.1863

[CIT0007] Liu B, Huang B, Hu G, He D, Li Y, Ran X, Du J, Fu S, Liu D. 2019. Isovitexin-mediated regulation of microglial polarization in lipopolysaccharide-induced neuroinflammation via activation of the CaMKKβ/AMPK-PGC-1α signaling axis. Front Immunol. 10:2650. 31798583 10.3389/fimmu.2019.02650 PMC6868066

[CIT0008] Liu-Bryan R, Pritzker K, Firestein GS, Terkeltaub R. 2005a. TLR2 signaling in chondrocytes drives calcium pyrophosphate dihydrate and monosodium urate crystal-induced nitric oxide generation. J Immunol. 174(8):5016–5023. 15814732 10.4049/jimmunol.174.8.5016

[CIT0009] Liu-Bryan R, Scott P, Sydlaske A, Rose DM, Terkeltaub R. 2005b. Innate immunity conferred by Toll-like receptors 2 and 4 and myeloid differentiation factor 88 expression is pivotal to monosodium urate monohydrate crystal-induced inflammation. Arthritis Rheum. 52(9):2936–2946. 16142712 10.1002/art.21238

[CIT0010] Lv H, Yu Z, Zheng Y, Wang L, Qin X, Cheng G, Ci X. 2016. Isovitexin exerts anti-inflammatory and anti-oxidant activities on lipopolysaccharide-induced acute lung injury by inhibiting MAPK and NF-κB and activating HO-1/Nrf2 pathways. Int J Biol Sci. 12(1):72–86. 26722219 10.7150/ijbs.13188 PMC4679400

[CIT0011] Lyu S, Ding R, Liu P, OuYang H, Feng Y, Rao Y, Yang S. 2019. LC–MS analysis of serum for the metabolomic investigation of the effects of pulchinenoside b4 administration in monosodium urate crystal-induced gouty arthritis rat model. Molecules. 24:3161. 10.3390/molecules24173161 PMC6749452 31480258

[CIT0012] Martin WJ, Harper JL. 2010. Innate inflammation and resolution in acute gout. Immunol Cell Biol. 88(1):15–19. 19935764 10.1038/icb.2009.89

[CIT0013] Mencherini T, Picerno P, Scesa C, Aquino R. 2007. Triterpene, antioxidant, and antimicrobial compounds from *Melissa officinalis*. J Nat Prod. 70(12):1889–1894. 18004816 10.1021/np070351s

[CIT0014] Mitroulis I, Kambas K, Ritis K. 2013. Neutrophils, IL-1β, and gout: is there a link? Semin Immunopathol. 35(4):501–512. 23344781 10.1007/s00281-013-0361-0

[CIT0015] Nonaka F, Migita K, Haramura T, Sumiyoshi R, Kawakami A, Eguchi K. 2014. Colchicine-responsive protracted gouty arthritis with systemic inflammatory reactions. Mod Rheumatol. 24(3):540–543. 24533551 10.3109/14397595.2013.874732

[CIT0016] Qing YF, Zhang QB, Zhou JG, Jiang L. 2014. Changes in Toll-like receptor (TLR)4-NFκB-IL1β signaling in male gout patients might be involved in the pathogenesis of primary gouty arthritis. Rheumatol Int. 34(2):213–220. 24036988 10.1007/s00296-013-2856-3

[CIT0017] Raucci F, Iqbal AJ, Saviano A, Minosi P, Piccolo M, Irace C, Caso F, Scarpa R, Pieretti S, Mascolo N, et al. 2019. IL-17A neutralizing antibody regulates monosodium urate crystal-induced gouty inflammation. Pharmacol Res. 147:104351. 31315067 10.1016/j.phrs.2019.104351

[CIT0018] Sabina EP, Rasool M, Mathew L, Ezilrani P, Indu H. 2010. 6-Shogaol inhibits monosodium urate crystal-induced inflammation—an *in vivo* and *in vitro* study. Food Chem Toxicol. 48(1):229–235. 19819286 10.1016/j.fct.2009.10.005

[CIT0019] Shi Y, Evans JE, Rock KL. 2003. Molecular identification of a danger signal that alerts the immune system to dying cells. Nature. 425(6957):516–521. 14520412 10.1038/nature01991

[CIT0020] Simon Taylor R. 2017. BET 1: prednisolone for the treatment of acute gouty arthritis. Emerg Med J. 34(10):687–689. 28963379 10.1136/emermed-2017-207129.1

[CIT0021] So A, De Smedt T, Revaz S, Tschopp J. 2007. A pilot study of IL-1 inhibition by anakinra in acute gout. Arthritis Res Ther. 9(2):R28. 17352828 10.1186/ar2143 PMC1906806

[CIT0022] van Walsem A, Pandhi S, Nixon RM, Guyot P, Karabis A, Moore RA. 2015. Relative benefit-risk comparing diclofenac to other traditional non-steroidal anti-inflammatory drugs and cyclooxygenase-2 inhibitors in patients with osteoarthritis or rheumatoid arthritis: a network meta-analysis. Arthritis Res Ther. 17:66. 25879879 10.1186/s13075-015-0554-0 PMC4411793

[CIT0023] Wang WC, Xia YM, Yang B, Su XN, Chen JK, Li W, Jiang T. 2017. Protective effects of tyrosol against LPS-induced acute lung injury via inhibiting NF-κB and AP-1 activation and activating the HO-1/Nrf2 pathways. Biol Pharm Bull. 40(5):583–593. 28190857 10.1248/bpb.b16-00756

[CIT0024] Zhang Q, Lenardo MJ, Baltimore D. 2017. 30 Years of NF-κB: a blossoming of relevance to human pathobiology. Cell. 168(1–2):37–57. 28086098 10.1016/j.cell.2016.12.012 PMC5268070

[CIT0025] Zielińska-Pisklak MA, Kaliszewska D, Stolarczyk M, Kiss AK. 2015. Activity-guided isolation, identification and quantification of biologically active isomeric compounds from folk medicinal plant *Desmodium adscendens* using high performance liquid chromatography with diode array detector, mass spectrometry and multidimentional nuclear magnetic resonance spectroscopy. J Pharm Biomed Anal. 102:54–63. 25240729 10.1016/j.jpba.2014.08.033

